# Drought in the Semiarid Region of Brazil: Exposure, Vulnerabilities and Health Impacts from the Perspectives of Local Actors

**DOI:** 10.1371/currents.dis.c226851ebd64290e619a4d1ed79c8639

**Published:** 2018-10-29

**Authors:** Aderita Sena, Carlos Freitas, Patrícia Feitosa Souza, Fernando Carneiro, Tais Alpino, Marcel Pedroso, Carlos Corvalan, Christovam Barcellos

**Affiliations:** Institute of Scientific and Technological Communication and Information in Health (ICICT), Oswaldo Cruz Foundation, Rio de Janeiro, RJ, Brazil; Center for Study and Research of Emergencies and Disasters in Health (Cepedes), Oswaldo Cruz Foundation, Rio de Janeiro, RJ, Brazil; Institute of Scientific and Technological Communication and Information in Health (ICICT), Oswaldo Cruz Foundation, Rio de Janeiro, RJ, Brazil; Health and Environment Department, Oswaldo Cruz Foundation of Ceará, Fortaleza, CE, Brazil; Center for Study and Research of Emergencies and Disasters in Health (Cepedes), Oswaldo Cruz Foundation, Rio de Janeiro, RJ, Brazil; Institute of Scientific and Technological Communication and Information in Health (ICICT), Oswaldo Cruz Foundation, Rio de Janeiro, RJ, Brazil; Sydney Medical School, University of Sydney, NSW, Australia; Institute of Scientific and Technological Communication and Information in Health (ICICT), Oswaldo Cruz Foundation, Rio de Janeiro, RJ, Brazil

## Abstract

**Introduction:**

The objective of this study was to understand and assess the perception of communities, organized civil society, health professionals, and decision-makers of several governmental institutions, regarding vulnerabilities and health impacts in drought prone municipalities of Brazil.

**Methods:**

This study was carried out through a qualitative investigation in eight municipalities in the Brazilian Semiarid region. Data collection was done through semi-structure and structure interviews, and discussion with local actors, which included communities groups, health professionals, governmental managers and organized civil society.

**Results:**

The results point to the local actors’ concerns and to the fragility of the health sector in the planning of integrated actions directed towards risks and impacts associated with drought conditions on human health.

**Discussion:**

The lack of a specific knowledge contributes to making invisible the process that determines the impacts of drought on health, leading to an acceptance of drought in those municipalities, reducing the capacity of the health system to respond to droughts.

**Keywords:**

drought, vulnerability, risks, health, perception, Brazilian Semiarid, resilience

## Introduction

**Emerging challenges of environmental changes**

Global environmental changes, including climate change are bringing new threats and challenges to environmental and human systems. A report of the Intergovernmental Panel on Climate Change (IPCC) indicates an increase in the global average temperature of the planet and changes in precipitation, with projections to get worse in the future 1^,^^2^. These changes will cause serious impacts on climatic and environmental conditions, and consequently, influence the economic, social and health conditions of populations, which will have consequences on human development and on the risk of disaster 2^,^^3^^,^^4^.

In the Semiarid region of Brazil, the greatest vulnerabilities associated with climate change are the difficulty of access to water in sufficient quantity and quality; the susceptibility to extreme drought events; impacts on food production and food security; and the multiple short and long term impacts on human health 2^,^^3^^,^^5^^,^^6^^,^^7^. The impacts of climate change on human health are diverse, and may occur through direct or indirect exposure and through changes on economic and social conditions of affected populations 8^,^^9^. Direct effects are deaths and injuries caused by extreme weather events, such as droughts and heavy rains, and extreme heat. Indirect effects are mediated by impacts on the environment, for example, infectious diseases transmitted by water, food and vectors, and respiratory diseases related to air quality in the short term. Non-communicable diseases such as undernutrition and mental stress may occur in the long term 8^,^^9^^,^^10^^,^^11^. The effects on social and economic conditions are associated to declining environmental conditions, which can compromise the access to health services, agricultural and livestock production, unemployment, insufficient income, and population migration. These conditions may cause or amplify health outcomes such as cardiovascular diseases, hypertension, mental illness such as depression, anxiety and stress 9.

The magnitude and extent of these impacts will depend on: 1) what is impacted (water infrastructure, agricultural production, ecosystems that affect the occurrence and transmission of diseases, economic development, employment); 2) who is exposed (individuals, communities) and; 3) existing vulnerabilities (political, demographic and health infrastructures, income, unsustainable practices, individuals physical factors) 4^,^^12^^,^^13^. The tendency of these effects is to affect poorer regions, which present greater social and economic vulnerabilities, difficulty to cope with and manage the impacts, and insufficient financial condition to implement efficient adaptation and mitigation policies 2^,^^4^^,^^10^^,^^13^^,^^14^.

All of these factors result in enormous challenges, and this is particularly the case of the Brazilian Semiarid region, which already presents a difficult situation of severe and recurrent droughts, a high proportion of populations living in poverty and other social and economic vulnerabilities.

**Drought impacts on systems and populations**

Drought is an extreme climatic event, which is characterised by the reduction of water reserves in a geographic area, precipitation below the normal average and a high rate of evapotranspiration influenced by increased air temperature 15^,^^16^^,^^17^. The types of drought, meteorological, hydrological and agricultural, reflect differences in both climatic characteristics and land use. These differences depend on local problems and needs related to agriculture, livestock and water resource management, which impact differently in the economic and living conditions of communities 17^,^^18^^,^^19^. Impacts on access to water and food can compromise systems and services, especially in poor regions, where there is low economic development 1^,^^20^^,^^21^^,^^22^^,^^23^.

The impacts of an extreme event tend to be amplified in a situation of multiple vulnerabilities. In the case of drought, especially for a prolonged period, local vulnerabilities include low income, living in rural or remote areas, low educational and socioeconomic level, family food production, other unfavorables environmental conditions, and low level of development 24^,^^25^^,^^26^^,^^27^. These conditions when added to meteorological and/or hydrological droughts contribute to the risk of agricultural drought with the possibility of affecting economic conditions and life of the communities 1^,^^28^^,^^29^^,^^30^.

From studies about the social problems that these all types of drought cause on the development of a region, the term socioeconomic drought was developed 17^,^^18^. According to Carvalho,^18^ socioeconomic drought is determined by the intensity of multiple impacts on a society, especially in its economic development, health and well being.

A clear example of this situation is pointed out by Castro 31 who explains that in past droughts in the Northeast of Brazil (referring to droughts in 1877 and 1932), the climatic condition together with an inadequate socioeconomic and political infrastructure left populations in worse conditions than in normal times. Droughts marked this region with death (of people and animals), hunger and devastation of the land. Faced with this situation people were forced to migrate to other places in search of a better living condition for their families, but this migration process brought other risks, both for the families left behind and for the migrants, which depended on environmental and social conditions of the new place.

Drought impacts continue today to be multiple and intense, having large repercussion on populations, which present higher socioeconomic vulnerabilities to cope with the adverse effects of drought. This situation results in and influences social inequalities and injustices, which in turn results in differences in livelihoods, human rights, health and human well being 28^,^^29^^,^^32^^,^^33^^,^^34^.

**Drought and vulnerabilities in the semiarid region of Brazil**

In Brazil, drought is considered the most frequent climatic event, reaching in greater proportion the area of the Brazilian Semiarid, with intense, prolonged and recurrent droughts 35. The Atlas of Natural Disasters in Brazil reported 19,517 registered events of drought for the period between 1991 and 2012, corresponding to 48% of the total of 39,837 records of all types of natural events in the country. The Northeast region presented most of the events of drought, as well as the highest number of deaths and affected people 36.

Considering the most recent delimitation of the Semiarid region in Brazil, carried out in 2017 by the Ministry of National Integration, this area increased from 1,135 municipalities to 1,262, occupying a territorial area of approximately 1,530 million km2 (18% of the Brazilian territory). The current area covers parts of all nine states of the Northeast region (covering a large part of the states of Rio Grande do Norte, Ceará, Piauí, Pernambuco, Paraíba, Alagoas, Bahia e Sergipe, and a small part of Maranhão), and the northern part of the Southeast region in the state of Minas Gerais. This all area has a population of approximately 26.5 million inhabitants that corresponds to 12% of the population of Brazil 37^,^^38^^,^^39^. The Brazilian Semiarid is one of the most populated semiarid regions in the world 24.

The projections of temperature increase and precipitation decrease for the next decades will result in days and nights of extreme heat. This situation increases the possibility of extreme droughts and greater impacts to drought prone regions 1^,^^8^^,^^40^^,^^41^. Studies of the evaluation of impacts of climate change on Brazilian regions and its biomes point out to this region as susceptible to having more frequent and intense droughts in future 2^,^^41^^,^^42^^,^^43^^,^^44^^,^^45^^,^^46^. According to Nobre 43 and studies carried out by the Brazilian Panel on Climate Change (PBMC in Portuguese), it is estimated for the Brazilian Semiarid region a reduction of up to 70% in groundwater recharge by 2050, and a reduction of up to 20% in the flow of reservoirs and rivers, which will result in multiple impacts, mainly on agricultural irrigation process 43^,^^45^. The availability of water in reservoirs usually corresponds to only 40% of their storage capacity 18. Reduction in water availability coupled with the existing process of aridity and drought, climate change impacts, and unsustainable human practices (forest fires, deforestation, grazing, monoculture, irrigation and groundwater exploitation) tend to intensify the desertification 1^,^^2^^,^^47^ and salinization processes,^48^ which have already begun in the Semiarid region of Brazil 25^,^^41^^,^^44^^,^^46^^,^^48^^,^^49^^,^^50^.

The combination of these factors works as a pressure on water supply and demand, a condition that may result in greater environmental damages. These processes, if maintained, can increase economic and social damage, and generate risks that can intensify the health-illness process, which further extends the economic impacts and social exclusion of the population 18^,^^29^.

## Methods

This study was carried out in eight municipalities of the Brazilian Semiarid region. Its objectives were 1) to identify and evaluate the knowledge and perception of communities, organized civil society, health professionals and managers of several governmental institutions about the vulnerabilities and impacts related to drought and human health; and 2) to learn how the health sector manages drought situations to reduce risks.

The methodology used in this study was based on the analysis of primary qualitative data, through interviews conducted in two municipalities in the state of Rio Grande do Norte (RN) and six in the state of Ceará (CE). We sought to include municipalities with different socio-demographic characteristics that were representative of the municipalities of these two semiarid states (Table 1).

Firstly, a pilot field study was conducted in the municipalities of Rio Grande do Norte state (Acari and Currais Novos) with semi-structured interviews. After evaluating this method, the interviews in the municipalities of Ceará state (Canindé, Itatira, Parambu, Quixadá, Quixeramobim e Tauá) were adapted to a structured type with data collected by a scale of agreement (Likert scale method).

Each participant was informed about the aims of the research and the interview methodology and signed a consent form authorizing recorded interviews. Participants were identified by a code for use on tabulating data, in order to protect the participant’s identity. This research was approved by the Research Ethic Committee of the Oswaldo Cruz Foundation.

**Characterization of the field study and interview groups**

The characteristics of the interviewed municipalities are shown in Table 1.

**Table:** **Table 1:** Indicators of eight municipalities in the Brazilian Semiarid region that participated in the research. Source: PNUD,^51^ based on IBGE data. Legend: Population – population of municipality; Illiteracy – Proportion of illiteracy population (%); Poverty – proportion of the population living in poverty (%); Water access – proportion of population with access to piped water (%); Under 5 Mortality – Under 5 mortality rate per thousands live births; Life expectancy – life expectancy at birth; MHDI – Municipal Human Development Index; IRIS – Drought Disaster Risk Index 52.

State	Municipality	Population	Illiteracy	Poverty	Water access	Under 5 Mortality	Life expectancy	MHDI	IRIS
RN	Acari	10958	18.9	20.8	87.5	23.4	71.7	0.679	33.7
RN	Currais Novos	42240	19.1	22.6	88.2	23.3	72.6	0.691	35.4
CE	Canindé	74224	27.0	45.2	73.7	25.7	70.9	0.612	44.5
CE	Itatira	18865	35.7	53.8	53.9	29.1	69.8	0.562	56.4
CE	Parambu	31257	38.0	51.6	63.7	25.8	70.9	0.570	52.3
CE	Quixadá	80117	24.9	36.2	72.1	23.8	71.5	0.659	44.0
CE	Quixeramobim	71409	26.4	38.4	78.7	21.6	72.3	0.642	44.8
CE	Tauá	55530	29.4	41.0	78.2	24.2	71.4	0.633	47.5

The indicators that represent the social (illiteracy), economic (poverty), environmental (water) and health (infant mortality) dimensions were selected because they are important measures of the development of a municipality or region. These indicators are also a measure of inequality. Life expectancy and MHDI are useful in analyzing human development and the improvement of life conditions in the municipalities.

MHDI considers three relevant dimensions: health (opportunity to have a long life), education (opportunity to access knowledge) and income (opportunity to have a decent life) 51. Regarding IRIS, an index developed to measure the municipal disaster risk of drought,^52^ it corresponds to a set of indicators of vulnerability (measured by level of education and poverty), threat or hazard (measured by the number of damage assessments for drought and incidence of drought) and exposure (obtained by the percentage of population living without access to piped water). The results of the selected municipalities showed a high risk of disaster from droughts.

In total, 53 interviews were conducted between Rio Grande do Norte and Ceará, with 103 participants. In Rio Grande do Norte 18 semi-structured interviews were conducted in November 2015, in the municipalities of Acari and Currais Novos. The three groups of actors who participated in this phase corresponded to a community, health professionals and interviews in mixed groups (Table 2), with the participation of 38 women and 30 men.

Interviews were addressed to a community living close to a dam (Açude Gargalheiras, in Acari). The community participants had a diversity of professions and activities, such as fishermen, farmers, retired people, housewives, community leaders, teachers and students. Regarding health professionals these included community health agents, nurses and health managers. Three group interviews were carried out: one in a Quilombola village (a type of settlement consisting of people of African descent) with the presence of the community leader and the population; the second one in a larger group with participation of community leaders, population, and education and health professionals; and the third with an organized social group, the Union of Rural Workers and Family Farmers.

**Table:** **Table 2:** Number of interviews per group interviewed in two municipalities of Rio Grande do Norte: Acari and Currais Novos.

Group interviewed	Number of interviews	Number of participants
Community	9	9
Health professionals	6	6
Interviews in group	3	53
Total	18	68

In Ceará, 35 structured interviews were conducted in April 2016, in the municipalities of Canindé, Itatira, Parambu, Quixadá, Quixeramobim and Tauá, with a total of 24 women and 11 men. The three groups of respondents corresponded to organized civil society, health professionals and government managers (Table 3).

Health professionals interviewed included community health agents, nurses, doctors and health managers. The health managers were secretaries of health, coordinators of Primary Care and coordinators of Epidemiology, Environmental and Sanitary Surveillance. Interviews also included managers of Planning and Budget, Agriculture and Livestock, and Water Supply and Sewerage System. Regarding the institutions of Organized Civil Society, they included representatives of the Union of Rural Workers and Family Farmers and Cáritas Diocesana de Crateús (a religious organization). These institutions integrate their activities with the support of a regional organization (Articulação do Semiárido) that puts in practice projects of coexistence with the Semiarid, through the support of public policies.

**Table:** **Table 3:** Number of interviews per group interviewed in six municipalities of CE state, Canindé, Itatira, Parambu, Quixadá, Quixeramobim and Tauá.

Group interviewed	Number of interviews	Number of participants
Organized Civil Society	7	7
Health professionals	14	14
Government managers	14	14
Total	35	35

**Data collection**

The interviews, both semi-structured and structured, addressed issues identified in the scientific literature. The questions for the structured interviews were improved based on the results of the semi-structured interviews conducted in Rio Grande do Norte. The questions were classified by relevant topics to facilitate the discussion. These topics were related to 1) social, environmental and economic vulnerabilities; 2) knowledge about drought and climate change; 3) effects of drought on human health; 4) factors or mechanisms resulting from the drought process that generate the impacts on health; 5) health sector interventions of preparedness, prevention and responses to reduce risks associated to drought; 6) evaluation and integration of actions, involving community participation; and 7) the resilience of population and government, including social programs.

**Data Analysis**

After collecting and organizing the data, the interviews (both, semi-structured and structured) were analysed by the method of analysis of content 53.

Data were organized by pre-defined thematic categories and sub-categories in order to facilitate the interpretation. Associations between elements was made, selecting the similarities, contrasts and differences; the understanding of the most relevant contents of the groups, in order to compare with the literature; and finally, an interpretative synthesis that answered both, the questions of the study and the formulation of new questions.

After organizing by topics, the data were analyzed in an articulation with the theoretical basis with the purpose of discussing the logic of meanings and the perception of the interviewees regarding the interface between drought, local vulnerabilities and impacts on health and on living conditions of the population who live in a semiarid climate.

For the analysis of Ceará data, the interview script consisted of a series of statements, following the Likert scale method, which contained six categories of response (totally agree, partially agree, partially disagree, totally disagree, do not know and does not apply). We first tabulated the responses within the six categories. Most of interviewees after responding to the statements within Likert scale, also expressed themselves on the subject. These statements were noted and considered in the analysis of the results. We transcribed the recordings and organized the data by thematic categorization, in order to facilitate the interpretation for content analysis, (as was done in Rio Grande do Norte).

Perceptions of the interviewees of both states, Rio Grande do Norte and Ceará (communities, organized civil society and government) were organized into four main thematic categories. Then, each of these categories and their corresponding variables were analyzed, including the perceptions of interviewees in both states. Key statements of some interviewees were highlighted.

## Results and Discussion

The results presented from the analysis of the eight municipalities of the states of Rio Grande do Norte and Ceará reflect the participants’ perceptions about four key areas: 1) socioeconomic and environmental vulnerabilities; 2) risk factors for diseases; 3) impacts of drought on human health; and 4) drought risk management (prevention, preparedness, response and recovery, including adaptation measures). Perceptions in this 4th key area refer to the 2nd, 5th, 6th and 7th topics related to the questions’ script described in the data collection section. These themes address strategies for preparedness and response for decision-making within the health system. It is important to clarify the difference between vulnerability and risk for the interpretation of responses. Vulnerability was understood as one of the conditions that make systems, populations and individuals more likely to be affected by a risk factor; and risks were understood as factors that can cause diseases 1^,^^54^^,^^55^^,^^56^. A similar scenario is observed between the two states. Table 4 presents the percentage of responses from Rio Grande do Norte.

**Figure:**
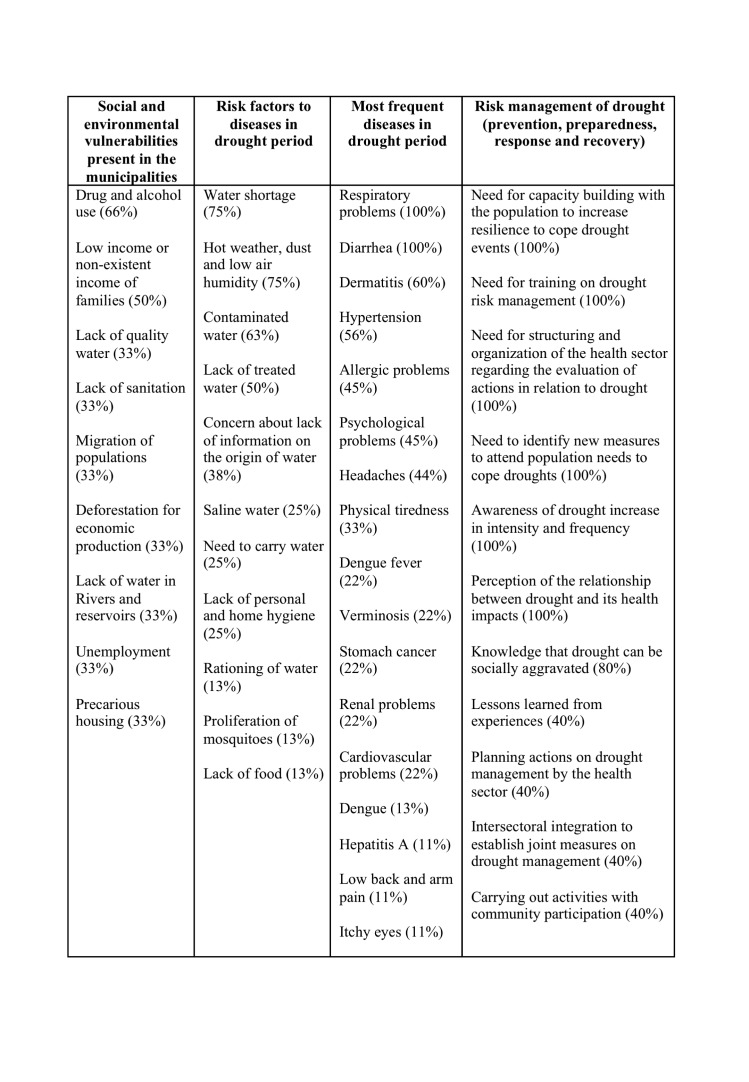
**Table 4:** Percentages of mentions in responses on the perception of the interviewees (communities, organized civil society and government) in relation to exposure to drought in the Rio Grande do Norte.


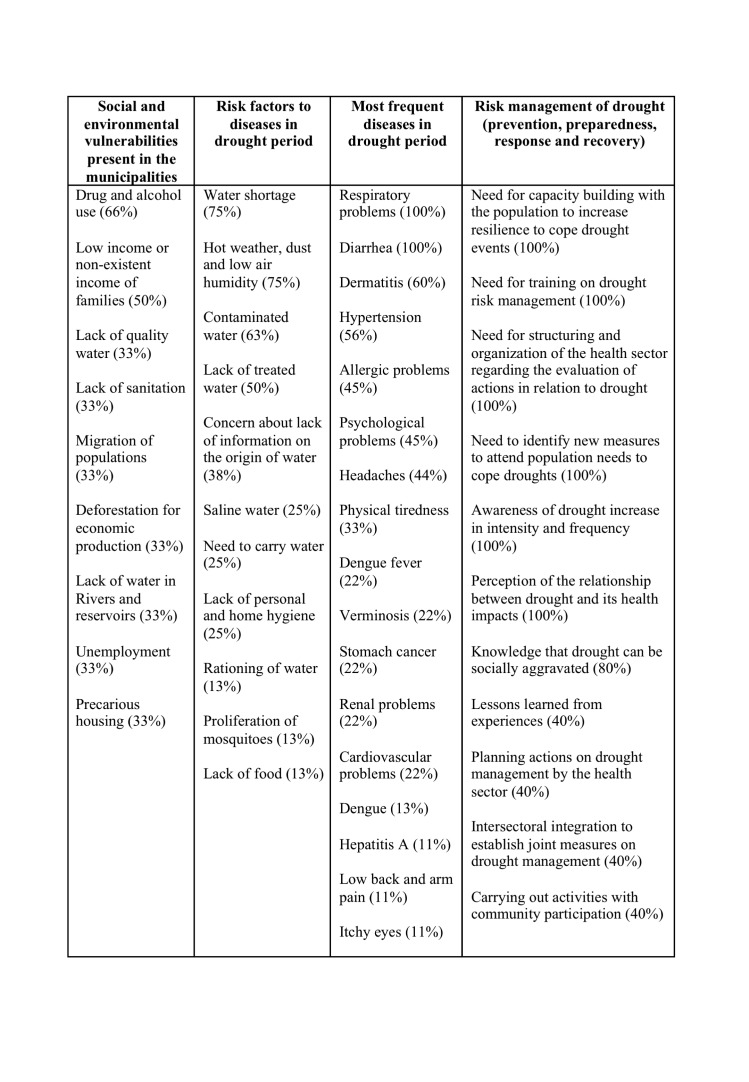


As for Ceará, Table 5 presents the percentage of agreement (total and partial) referring to the Likert scale, for each one of the variables selected in the questionnaire.

**Figure:**
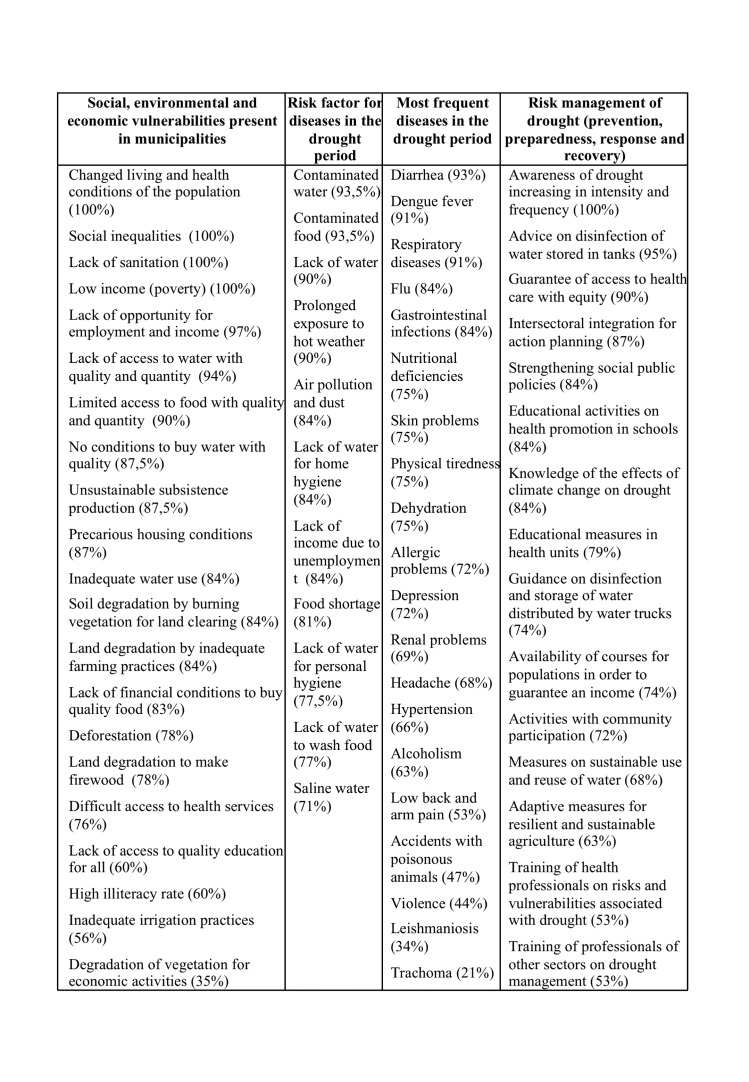
**Table 5:** Percentage of agreement (totally and partially) of the interviewees in Ceará regarding perceptions of exposure to drought.

**Social, environmental and economic vulnerabilities present in the municipalities**

In general, the vulnerabilities mentioned by participants reflect the unfavorable situation of populations living in the Brazilian Semiarid. It has been reported that despite the improvements in this region due to governmental social programs, there is still poverty especially in rural areas. The living and health conditions of the populations are changed by the magnitude of the drought impacts, mainly due to their low income and lack of access to employment, which increases social inequalities.

Many interviewees mentioned the low economic development in these municipalities and also low agricultural and livestock production for business purposes. These social and economic vulnerabilities coupled with the most mentioned environmental vulnerabilities, such as lack of sanitation, lack or scarcity of water and access to food in quantity and quality, as well as precarious housing, especially in rural areas, can increase the existing social inequalities in the region, as pointed out by several studies 57^,^^58^^,^^59^^,^^60^^,^^61^. Some statements express this problem, such as those mentioned by the managers regarding social inequalities and poverty, and by health professionals regarding the precarious housing and sanitation conditions. We highlight some statements: ‘Inequalities exist, service structures and poverty can already be mapped’. ’In the periods of drought people get poorer by affecting their economic conditions and their mental health’. ‘Housing conditions are inadequate, many homes do not have a safely managed sanitation service, nor sewer, and many people live in the same house together’.

Regarding the lack of access to water, most interviewees reported that even with some initiatives by the government to establish measures for access to water, the access is still limited. This situation results in the need for rationing water. Examples of such initiatives include: the construction of dams, tanks, cisterns, fountains, deep wells, and distribution of water by water trucks, during long periods of droughts. It causes water supply shortages in some communities (both through the water tanks and water distributed by truck), increasing their financial expenses due to the need to buy water, which is more expensive in drought periods. It also increases the risk of diseases. The following statements mentioned by health professionals express this situation well: ‘Piped water only comes every 15 days, forcing people to buy water or store it’. ‘When there is no water supply, the population looks for alternatives, which can bring complications to health’. Regarding the lack of financial conditions to buy water, the following divergent statement is highlighted: ‘Most people have condition to buy water and those people who do not, receive water distributed by the government’.

It was reported that even with water scarcity, there is still waste, especially in urban areas, and that sometimes it occurs by inadequate water use by family farming, through cisterns and agricultural productive yards. The interviewees also stated that the practice of flooding is used to irrigate the agricultural production, which requires a large amount of water, arguing that there is no technical training or improvement of sustainable practices that can improve production in drought prone areas.

When water was associated with lack of quality for human consumption, it was considered that there is no adequate treatment of water by the government, and that there is inappropriate storage, mainly by the population. On the other hand, there were divergences pointed out by the interviewees. Some participants mentioned that the water in the wells has poor quality, because it is saline, and others said that it was of good quality. Regarding water distributed by trucks, some interviewees stated that this water is treated and has good quality, but others said that it does not have quality. The following statements by some managers and health professionals express this question: ‘The water in the dam has poor quality and the water in the cistern is good’. ‘Most of the reservoirs are contaminated’. ‘There is water supply, but with inappropriate quality’. ‘The water distributed by the government is treated, but the water that we buy, we do not know if it is supervised’.

In relation to inadequately stored water by the population, a manager explained that: ‘The populations store water and do not let the professionals, who are responsible for control of zoonoses, do the right work in the houses, which can result in people getting sick with arboviruses. There were also reports that in some communities people use the water from reservoirs to bath and do home hygiene, even though the water is dirty and contaminated; as one farmer stated: ‘Any water, dirty or not, is useful for something, and it has to be used’. It is observed through these statements that the lack of access to water, especially fresh water, may result in inadequate practices of using water without quality. These situations reflect that people exposed to drought conditions take advantage of any opportunity of water availability; even knowing that water is not suitable for human consumption.

In relation to limited access to food in quality and quantity it was pointed out, mainly by health professionals that this condition is more related to social inequalities and limited knowledge about food quality. The following statements express this: ‘Access to quality food depends on each family priority. It is necessary to educate and make people aware about food quality’. On the other hand, most organized civil society has reported that the government’s social programs have significantly improved access to food, although some families, who really need it, do not receive these benefits and sometimes they go hungry. In general, most health professionals said that there were more obese children than undernourished.

Regarding the lack of financial condition to buy food, the responses showed a similar perception. They stated that in spite of the increase in food prices and the lack of employment, people do not starve as they used to during long drought periods before. This is due to a social program to help families in need (called Bolsa Família, a government program of income transfer, which has some conditionality that strengthens access to basic social rights such as education, health and social assistance) and also because of benefits from other social programs, which have improved family access to food (despite inadequate eating and food purchasing habits). This social program aims to reduce poverty, especially in the poorest regions, and to help families to overcome existing vulnerabilities 62. Other government benefits which subsidize improvement in the development of the Brazilian Semiarid region are: Cistern Construction; Food Acquisition Program; National Family Farming Strengthening Program; National School Feeding Program; Government support to loss of agricultural produce due to drought; Drought assistance; Rural Activities Program; and National Program of Inclusion of the Young, which helps in the professional formation of young people of low income.

Regarding to lack of access to health services, despite high percentage of agreement, it is important to refer to the observation that there are health services, including specialists and activities by the Family Health, but sometimes there are difficulties accessing these services. Statements from interviewees from the organized civil society were highlighted: ‘There is no lack of health services, but there is a need to improve the infrastructure of these services in terms to guaranteeing better access to whose living in remote communities, far from the health district’s headquarters’. ‘There is also a need to improve the implementation of the Family Health program for all families, because this program does not yet include 100% of the rural families, who need assistance’. We also highlight the statements from group interviews: ‘Service provision here in the community only occurs every two months and only 15 people can be seen, one per family’. ‘In early years there were more children deaths, recently the access to the hospital is better, avoiding deaths’. It was reported by the organized civil society that despite the difficulty of access to health care services, the Unified Health System (in Portuguse, Sistema Único de Saúde – SUS, the universal health system in Brazil) represents the only means of health care for a large part of the population. It is considered a health reference for them. In the group interviews, it was mentioned that although the SUS is for everyone, in rural areas coverage is not complete.

Ebi and Bowen 61 point out that health services should not be interrupted, at the time when their demand may increase, as for example, in periods of drought. The statements of a health manager express this situation well: ‘Due to the lack of piped water, having water only in deposits or tanks, the health officers cannot act, increasing concerns about disease’. ‘When there is no water, the dental service is affected; and there is no water for the vaccine room, compromising hand hygiene’. ‘Health units have had to close due to lack of water’.

In relation to the lack of access to quality education for all, we highlight important observations. The first concern refers to quality education, especially in rural areas. Most of the organized civil society indicated that education needs to be improved. It needs to be expanded to all grades, in order to avoid student displacement to others cities, often distant, and having nothing to eat. The second concern, pointed out by both organized civil society and managers, was about the need for education to be focused on environmental issues, especially on local issues related to the semiarid region, as demonstrated by the following statements: ‘It is necessary to have an education focused on a rural area vision, including training and values, not an education only with urban vision, as the traditional education is’. ‘Teachers need to learn and have specific training to work on the reality of the students whose living in the rural areas’. Regarding to the high illiteracy rate in the semiarid region, the interviewees claimed that there is more illiteracy in the elderly, and that it is not a high rate. Currently there is a government program called Education for young people, adults and elderly, allowing access and opportunity of education for all.

We also highlight an important manager’s comment about the need for access to schooling: ‘We need to strengthen schools, because when there is a lack of food, it affects the students’ performance. The school lunch is the only food supply, and sometimes the schools are closed because there is no water’. This perception also shows how it is important that the basic services have uninterrupted access to water. In the case of schools, in addition to the provision of food, it allows for better children cognitive development, and also a great value to parents by being reassured that their children will have an opportunity to eat.

Regarding questions about land degradation it is observed that many tendencies are confirmed by indicators, such as production of firewood, deforestation, inadequate crop practices, and burning vegetation to prepare the soil for crops. Concerning the production of firewood, the majority claimed that there are communities that still use wood stoves, produce firewood to sell, charcoal preparation for sale, and the use of coal for ceramics production in some localities. Regarding deforestation, most of interviewees confirmed that this practice still occurs, but that it has been decreasing with the Family Agricultural program, and with the inspections by the Government Environmental Agency. An important observation was made by the organized civil society in this statement: ‘When addressing environmental degradation it is unfair to compare a small family production farmer with a big businessman’. The perception on these issues is also consistent with the following statements related to the increase of drought, both in frequency and intensity, and related to climate change. Almost 100% of the interviewees agreed and attributed as causes of environmental changes anthropogenic activities, such as soil degradation, deforestation and natural resources exploitation. We highlight an interesting observation made by a health professional: ‘I am 46 years old and the situation is getting worse, the rains are scarcer, rivers used to fill when it rained, currently it does not happen’. All the issues identified above, have been identified in the literature 1^,^^2^^,^^12^^,^^41^^,^^44^^,^^46^^,^^47^^,^^49^^,^^63^.

Regarding the groups considered most vulnerable to the impacts of drought, all the interviewees agreed with the identification of the elderly, children and pregnant women, as pointed out by the literature,^24^^,^^25^^,^^26^ but emphasised that the most vulnerable group are rural farmers, especially, elderly farmers. In relation to health care services, several interviewees expressed that health care should be the same for all, and not differentiated between specific social or population groups.

It is important to highlight the occurrence of migration mentioned by health professionals and managers, such as in the following statements: ‘Lack of employment and income leads the process of migration from rural to urban areas, causing depression and concerns.’ ‘Drought causes migration, many families go to other states and sometimes come back because it did not work for them; others go and cannot come back, living marginalized’. ‘There is no agriculture, so people leave the countryside for the cities to search for a better life’. ‘With these five years of drought, the production of subsistence has become unfeasible; those who have a better level of education migrate to the cities, because they no longer want to work in the fields’. ‘The rural exodus is increasing and the peripheries [referring to the urban zone] are growing more and more’. What is perceived in these different statements is the reflection of both the magnitude of drought impacts in the current period [referring to the 2011-2016 drought period] and the fragility of the local infrastructure, still inadequate to keep farmers in the rural drought prone areas. Another perception referred to these statements is the persistence of inequalities in this region, which may influence the migration process contributing to people maintain or entering the cycle of poverty, with a greater possibility of being exposed to other risks and vulnerabilities in the places to which they are migrating.

**Risk factors for diseases in drought periods**

In general, the perception about risk factors during droughts periods, presented a strong association with the most prevalent diseases during drought.

The following statements from health professionals express well the perception that lack of access to water or the scarcity of water, and water without quality, are direct risk factors of diarrhoea and gastrointestinal infections. Also these risk factors can indirectly generate other risk factors that determine illness. Examples include difficulty in maintaining hygiene, lack of food production, food contamination, difficulty finding employment and income, and the concerns regarding all of these factors, in their life 11^,^^29^^,^^61^^,^^64^^,^^65^. For example, regarding contaminated water: ‘The major complaint and population dissatisfaction is the lack of treated water’. ‘Lack of piped water causes concern about its origin because it can cause illness’. ‘There was no looting because of the drought, but there were 30 deaths because of contaminated water’, noting that in the past, desperately poor people looted stores for food. The health professionals also mentioned: ‘In the periods of drought, dirty water brings Hepatitis A’. ‘We had outbreaks of Hepatitis A in 2012’. As for hygiene issues it was mentioned: ‘With the lack of water, it is impossible to maintain hygienic practices, both individually and in the house’. ‘People depend on the water truck, which is of low quality limiting the use for body and housing hygiene’. ‘With lack of water, there is no way to have proper hygiene’. Regarding food and nutrition: ‘Due to lack of water serious health problems can arise related to insufficient food and even to personal hygiene’. ‘The lack of water reduces the supply of food because there is no more production and the prices of food increase’. From a health manager perspective: ‘The greatest fear and concern of people is the lack of water because they can loose their animals due the lack of grazing, and because of diseases’.

As for the inexistence or difficulty of obtaining an income and the opportunity of employment, the following statements express the severity of this risk factor, associated principally with psychosocial problems: ‘People suffer with unemployment’. ‘The cause of the lack of employment is that there is no economic development in the municipality and region’. ‘Drought causes unemployment that leads to family disruption’. These statements can be summarized in the following conclusion by a health Professional: ‘Drought is socially worsened by the precarious political and economic structures. If there were a political project and more opportunity for employment and income, the impacts of drought would be less’.

Some health problems, such as respiratory diseases, allergies, cardiac problems, flu, diarrhea and dry skin were attributed to prolonged exposure to the sun, hot weather and low air humidity. This perception is corroborated in some studies 11^,^^66^^,^^67^. To this concern we highlight statements that have been mentioned by two group interviews: ‘At night people who have good conditions sleep better than those who don’t, because dryness and heat can affect more those who cannot afford to buy a fan’. ‘The very strong sun is causing headache and tiredness, leaving people sick’.

Although concerns about saline water was mentioned by a few interviewees, it would important to consider further investigations, given the association of this risk factor with some diseases mentioned by the interviewees, such as hypertension, skin problems and renal problems, as well as the increase of hemodialysis procedures in some drought prone municipalities. This perception can be seen in the following statements: ‘Search for water is increasing, compromising the groundwater and water sources. The water is drawn from the local crevices, which is salty’. ‘The water is salty, which can bring skin problems’. ‘There are renal problems due to the lack of water and having salty water’. ‘There is hypertension, and renal and heart problems due to salty water’.

**Most frequent diseases during drought period**

The most common illnesses perceived by the interviewees during drought periods were diarrhea, respiratory diseases (respiratory problems and flu), dengue and gastrointestinal infections. Some statements related to this perception stand out: ‘Lack of water or contaminated water cause belly pain and diarrhea’. ‘The water from the water truck is for drinking, but it causes pain in the belly’. Although diarrhea had been cited as the most frequently illness, it was reported to be very common both during periods of drought, as well as during rain periods. This statement calls the attention to factors related to the lack of quality water for human consumption, food safety due to contaminated water and food, and the lack of water for personal hygiene. Sanitation is an important element in reducing several gastrointestinal infections including diarrhea, which can also be intensified in with higher temperatures. Several studies corroborate with this perception 11^,^^29^^,^^64^^,^^65^^,^^68^^,^^69^. Regarding respiratory problems, the following statements from health professionals reported the perception related to prolonged sun and heat exposure, and to low air humidity: ‘It is during the hottest and least humid period of the drought, that respiratory problems appear’. ‘Around where I live the most frequent problems in the population are respiratory’. ‘There is a lot of hospitalization from diarrhoea and lung problems with cough, due to low air humidity’.

Although most of the interviewees agreed that there are cases of dengue during drought periods, it was mentioned that this disease also increased during the rainy season. A relevant factor to be considered, according to literature, is the persistence of Aedes aegypti eggs in the dry season, due to the easy adaptation of the mosquito to the human environment 70. This situation tends to be aggravated in places with structural problems of water supply, facilitating the vector production through natural or artificial reservoirs.

As for physical fatigue and headache, it was mentioned that these problems are associated with work exposure to the sun and the hot and dry climate. The cases of dehydration were associated with some disease that causes diarrhea. It also was mentioned that these illnesses are well controlled by health system, because all cases when identified are immediately referred for treatment with oral rehydration and also to the Food and Nutrition Program. Regarding skin and allergic problems, most interviewees stated that skin problems are associated to hot and dry weather. Some have reported that some families use the water from the reservoirs to bathe and wash clothes, even knowing that the water is dirty, which could also result in allergic problems.

Most interviewees agreed that there are no cases of undernutrition, but there are cases of nutritional deficiencies in low-income populations. This occurs even among the families who receive financial benefits from the government, although this situation has improved greatly, as shown in the study by Rasella et al. 71. It was mentioned that cases of nutritional deficiencies occur due to some factors such as, limited knowledge about food quality; preference for the most practical and cheapest food; or consumption of those foods influenced by the media. As previously noted, there were more cases of obesity than undernutrition, which warrants further investigation. We also note that the literature points out important effects caused by nutritional deficiencies, such as infections. For example, when infections are added to other vulnerability factors, such as food shortage and poverty, it can bring about serious consequences, mainly to children and pregnant women 71^,^^72^.

As for depression, most organized civil society and health professionals agreed there were increased cases of depression during drought periods, even though they had never noted this association before. Some have reported knowing cases of depression or anxiety, including suicide in older farmers for several reasons such as loss of agricultural production, loss of animals, idleness, worries about not being able to sustain their family, sadness and anguish. However they were not certain of the association between depression and suicide with problems caused by drought. The following statements express this perception: ‘We don’t know if it is depression, but we see sadness in rural men’. ‘The lack of employment and income brings depression and worry to the rural man’. ‘I know cases of depression in farmers who see their animals dying, but I had never thought that it could be associated with drought’. ‘Depression has become part of population’s life who live in rural areas, it is very common here’. ‘There are many cases of suicide in elderly people, but we do not know if the reason is associated to drought’. ‘There are cases of suicide due to depression and concern’; ‘There are farmers who committed suicide because they had lost everything’. ‘There are cases of suicide due to economic problems’. Other interviewees referred that the cases of depression have a hereditary origin, because there were some cases of depression and suicide in the same family. All of this calls for a better investigation in this region, because the literature evidences the association of these mental health problems in drought prone areas, as for example, in the United States and Australia 73^,^^74^^,^^75^.

Concerns about the possibility of loss of agricultural produce and lack of food production to support the family and feed animals, and lack of income were also mentioned as significant problems for mental health. Both health professionals and a group interview show this perception according to the following statements: ‘Lack of access to water causes psychological problems, anxiety and concern about the elderly and the impacts of drought’. ‘More money is being spent to buy water than food, and this is causing psychological problems’. ‘Lack of employment and income affects the mental health of people who live in the rural areas’. These concerns were also attributed as a cause of high blood pressure.

As for alcoholism and violence, it was reported by most of the interviewees that there is no association with drought, stating that the cause is due to low income or financial difficulties. However, the literature identifies this risk 77.

Regarding to pain in the lower back and arms, it is important to note that the state of Rio Grande do Norte pointed this out due to the need for people to carry water from wells and fountains, often for long distances. According to those interviewed in Ceará, this problem occurs more in older people as a consequence of previous droughts, but today few people need to carry water because the conditions of water supply and access have improved.

Accidents with venomous animals and leishmaniasis were hardly mentioned, despite the latter being informed by the literature 78. Regarding trachoma, although the answers pointed out little knowledge about the subject, especially, whether there is an association with drought, it was reported that in that same period of the survey, a study was being carried out in schools to diagnose trachoma, which resulted in some positive cases. This bacterial infectious diseases deserves a better investigation in that area, because the literature points out that the main risk factors are associated with lack of hygiene and lack of sanitation. This is a prevalent disease in Brazil, with medium and high endemic levels in the Northeast, mainly in the poor municipalities with low socioeconomics status,^79^^,^^80^ characteristics present in the semiarid region.

The majority of interviewees’ perceptions about the impacts of drought on human health agree with the literature in identifying risk factors arising from damages in the systems and services and the vulnerabilities present in the region, as shown in Table 6. The symbol (*) highlighted in the table refers to the diseases, injuries and mechanisms that affect the process of health determinants that were mentioned by the interviewees.

**Table:** **Table 6:** Possible impacts of drought on human health, through damages in the essential basic systems and services, and mechanisms for social determinants of health. Source: Adapted from references 11^,^^27^^,^^29^^,^^61^^,^^68^^,^^73^^,^^77^ * Diseases, injuries and mechanisms for health determination mentioned by the interviewees.

Damages in systems and services	Mechanisms of social determinants of health	Impacts on human health
Availability and safety of water	Water shortage*. Implication in irrigation for agricultural production and in livestock and fishing increasing the possibility of food shortages*. Consequences of water quality (non-potable water, saline water)*. Contamination of water by various means, such as algal blooms, bacteria, fungi, viruses. Contamination of food*. Damages to the functioning of the health services, with consequences to the provision of some sanitary procedures*. Consequences on the water supply and distribution system (for piped water, water trucks, cisterns, artesian wells, dams and other alternative sources)*. Need for household water storage, which may compromise water quality*. Difficulty in maintaining personal, food and home hygiene*. Rising water prices due to scarcity and high purchase demand*. Consequences of urban sanitation and sewage services. Change in vectors, hosts and reservoir cycles.	Gastrointestinal infectious diseases (diarrhea*, hepatitis A and other infections). Dehydration. Parasitic infectious diseases (worms*). Bacterial infectious diseases (trachoma, gastroenteritis). Dermatological infectious diseases*. Diseases transmitted by vectors and zoonoses (dengue*, zika*, chikungunya*, leishmaniasis, leptospirosis). Non-communicable diseases (hypertension, renal and mental problems)*. Infectious diseases transmitted by physical contact (flu*, conjunctivitis).
Availability and safety of food	Deficiency in agricultural, livestock and fishery production causing food shortages*. Difficulty in the sustainability of family agriculture, livestock and fishery*. Consequences in food quality and safety*. Food contamination*. Rising food prices*. Decreased access to food, especially to healthy food*.	Nutritional deficiencies*. Anemia. Malnutrition and its complications (low physical and cognitive development, deficiency of the immune system). Infections from food contaminated by viruses, bacteria, fungi, parasites (diarrhea*, cholera, hepatitis A*, worms*, other infections). Chronic non-communicable diseases* (hypertension, obesity)
Air quality	Low humidity*. Increased temperature (heat)*. Dust*. Contamination of the air by particles from fires, and toxins accumulated in soil and water.	Acute respiratory diseases (flu*, sinusitis, rhinitis, bronchitis, pneumonia). Allergic respiratory diseases (asthma, allergic rhinitis)*. Diseases caused by fungi, viruses, bacteria
Cleaning, hygiene and sanitation	Difficulty in cleaning and hygiene (personal, household, water truck supply, food, health service equipment) due to lack of water*. Consequences of sanitation services, urban cleaning, health services* and other basic services.	Dermatological infectious diseases*. Parasitic diseases (worms*). Infectious diseases transmitted by viruses, bacteria, fungi (flu*, conjunctivitis, pneumonia, gastrointestinal infections*, hepatitis A*, trachoma).
Social and economic factors	Loss and damage in economic, livestock and subsistence plantations due to the difficulty in accessing water*. Loss or lack of employment and income*. Migration of populations seeking improvement in their quality of life, needing to face other social changes and cultural changes*, and changes in the epidemiological profile of the receiving areas. Displacement of the spouse to other municipalities in search of employment to supply family needs, which cause disruption and changes in the family structure and dynamics*. Loss of social identity. Uncertainty and concerns for the future*.	Psychological disorders (anxiety*, stress*, behavioral change generating other problems such as violence, alcoholism). Depression*. Suicide. Chronic non-communicable diseases (heart, hypertension)*. Increased demand of health services and other social problems in the places where people migrate to.
Health care services	Risk of interruption of health care procedures due to lack of water or contamination due to lack of hygienic conditions (dressing wounds, immunization, dentistry, and hospital services etc.)*. Increased demand for care and supplies of health services*. Risk of impacts in energy supply, impairing the use of health equipment, refrigeration of medicines and vaccines, and the health care of some hospital services.	Communicable and non-communicable diseases*. Mental disorders*. Allergic respiratory diseases*. Nutritional deficiencies*. Lack of or reduction of health care due to lack of working conditions, which may worsen the health conditions of the population*.

**Risk management in drought (prevention, preparedness, response and recovery)**

Interviewees in both states showed awareness about drought increasing in intensity and frequency, citing deforestation as a cause. A high percentage had knowledge about the increasing effects of climate change on drought, although most of those interviewed did not know how this process occurred. The following statements refer to these perceptions: ‘Drought is increasing because of deforestation, and degraded soil that does not hold water’. ‘Drought is increasing because of the natural and environmental factors influenced by anthropogenic actions on deforestation and pollution’. Among the actions related to drought risk reduction management, the largest percentage referred to having information on disinfection of water stored in cisterns, and that the population does not perform the procedures correctly. It was also reported that there are actions to guarantee access to health care with equity by the Family Health Strategy and there is intersectoral integration for action planning. Despite this result, most responses stated that the actions are not specifically planned for drought and health management. Some actions are integrated in specific issues, for example, the control of dengue, which requires integration between epidemiological and environmental surveillance, specifically with endemic diseases vector control agents. The following statements from health professionals express this: ‘We live with the problem, but there is still no focus to address it, we only stay in the direct medical care to the patient’. ‘We establish goals and our focus is related to health problems associated from other causes and not from drought’; ‘We made evaluations, but they are not adequate for the current reality of drought’. ‘There is no drought-related health planning policy’. ‘I have been working on health for eight years, there is a trained team, but I have never participated in any drought-oriented planning’. ‘There is a lack of integration between levels of the government [municipal, state, federal]’. ‘There is a need for more integration between sectors and more professional training in drought’.

Regarding activities carried out with community participation, none of the interviewees reported on exchange of knowledge and decisions among the population and professionals, regarding drought. Such initiatives would help increase resilience in the population and government 56^,^^81^^,^^82^. The interviewees informed that integration between the health sector and the population occurs only in specific issues related to government programs, for example campaigns required by the federal or state levels of government, but these do not address drought. Example of campaigns were: guidance on using sodium hypochlorite to treat water for human consumption; breast cancer prevention; combating dengue; vaccination campaigns; and actions related to national programs of control of high blood pressure, diabetes and monitoring of acute diarrheal diseases. The following statements express this perception: ‘The health sector does not do activities with the population to discuss about drought, because there is no knowledge about it’. ‘We only call the population in relation to breast cancer prevention campaign and for other campaigns demanded by the federal level’. ‘There is a group of people receiving health care, but there is no discussion about drought’. ‘In the past [referring to the period before the drought] we discussed about problems such as hypertension and diabetes, but not problems focused on drought’.

Considering droughts before the current one (from 2012 to 2016), most of the managers interviewed agreed that the public policies have improved and strengthened through changes in the government program of access of water and food, access to health services, education and sanitation, as well as the implementation of other social policies. Most of the interviewees reported that current public policies, especially the income transfer program Bolsa Família, has been contributing to better access to food, as shown in the following statements: ‘People do not go hungry like they did in past droughts, and there are no more looting for food because the program Bolsa Família helps families to have better access to food’. ‘The drought of 1980s was lower than now, but the impacts were greater, for example, there was distribution of poor quality food, there was no water policy, there were many diseases and there were many cases of looting. Nowadays, with social policies, there are more opportunities with the implementation of cisterns and the stimulation of production and trade have increased greatly’. However, most of the interviewees form civil society stated that water access policies need to be consolidated in permanent and preventive policies rather than emergency policies as they currently are. The following statement expresses this perception: ‘It is necessary to take actions in the Semiarid region, not as an emergency policy, but as public policy with financial resources’. Regarding to the policies of social programs, this statement stands out: ‘These policies need more supervision and better structure, both to avoid inadequate use of its benefits and to ensure that all families that really need these benefits are registered in the program. There are many families in extreme poverty that are not registered’.

Although most of the interviewees pointed out that educational measures are developed both in health units and in schools, through the School Health Program, they informed that they had never worked specifically on the issue of drought. The following statement of a civil society interviewee expresses this concern: ‘There is a project of continuing education that should enable secondary school teachers to focus on the local reality’, referring to the rural areas, including those affected by drought. ‘Many educational policies are developed only in the districts (referring to urban area), it should be decentralized to the rural communities’.

Regarding the implementation of adaptive measures for implementing a resilient and sustainable agriculture, all interviewees of organized civil society reported that the government sells a seed that is more resistant to pests, but the farmers prefer the seed called “crioula” (autochthonous seed, defined by them as the original seed of the land), which is more resilient to the weather and type of soil (i.e. the Caatinga biome), and has the opportunity to sustain future resilient plantations. The follow statement corroborate with this perception: ‘The seed we buy from the government dos not give pests, it must be because it has pesticides, but we have to buy every year, sometimes we can use it twice; for this reason we prefer the “crioula” seed, which can be planted several times’.

In relation to training the population with provision of courses to generate employment and income it was mentioned that the government together with the civil society offer several courses, but that often the population does not participate for lack of information. Regarding the training of health professionals on the management of risks and vulnerabilities associated with drought, most of them said that there is training in several issues, which may be associated with drought, but in relation to drought specifically, they had never received any training. Health professionals made the following statements: ‘It is necessary to have a training policy for health professionals to know how to relate diseases to the drought season’. ‘It is necessary to have more lectures, orientations, qualification and training of professionals to improve their knowledge to be able to orient the population’.

The results related to risk reduction management of drought (with measures of prevention, preparedness, response and recovery, including adaptation measures) by health professionals showed lack of organization and preparedness to reduce risks and vulnerabilities associated to drought. The information and communication process necessary for understanding and addressing the social determinants of health (taking into account the people, their beliefs, their cultures, their ways, their place where they live and their needs) is also neglected; not only diseases related to drought. Figure 1 summarizes the perception of populations and government regarding drought, in two states of Rio Grande do Norte and Ceará. It is highlighted in this figure the summarized results in around key issues, such as inequalities in the social vulnerability, poverty in the economic vulnerabilities, water in the environmental vulnerabilities; and the actions that were identified as necessary to increase the resilience of populations and health systems exposed to drought.

**Figure:**
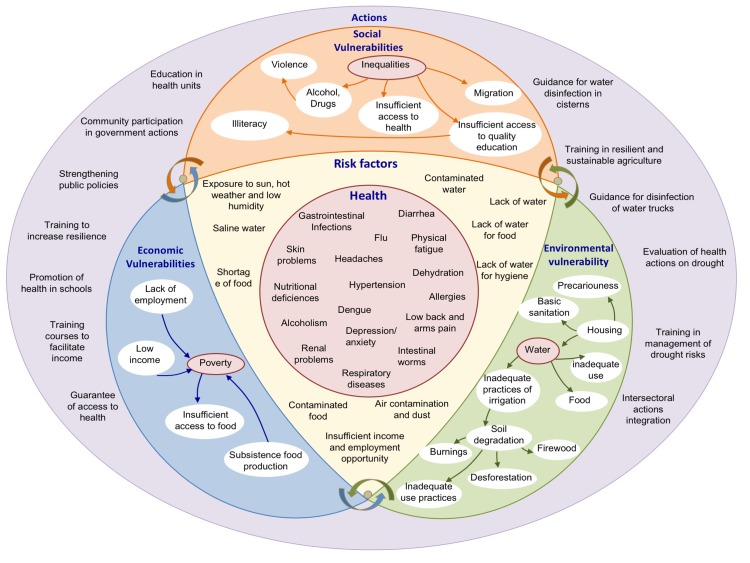
**Fig. 1:** Synthesis of the perception of the government and populations exposed to drought in the states of Rio Grande do Norte and Ceará.

## Conclusion

The results obtained from this research shows, in general, the fragility in the social and political infrastructure necessary to improve the living conditions of populations, particularly those that depend on family agriculture in drought prone areas. The conditions of social, economic and environmental vulnerabilities presented in the region can be amplified by the drought process, and can aggravate the impacts resulting drought events, thus disadvantaging the population of this region, as shown in the Fig. 1.

The general perception of the interviewees of the health sector shows an agreement with what is found in the literature on the relationship between drought and health conditions. However, the lack of specific knowledge regarding the relation between drought and health, both by health professionals and managers, and the lack of intra and intersectoral integration, prevents the appropriate planning of actions aimed at managing risks and impacts of drought on health. This fragility of the health system tends to disregard the process that determines drought conditions, with consequences in the recognition of this climatological event as a public health problem. Lack of comprehensive recognition of the issue may result in the possibility of greater negative impacts on health conditions and, consequently, on the life of these populations.

With the projection that droughts in this Brazilian Semiarid region would be aggravated in the future by the global process of climate change, there is a possibility to further increase unfavorable conditions for the populations. This would occur mainly due to climatic variability in the region, added to the social and economic vulnerabilities present in the area. From this point of view, there should be a greater concern to subsidize the formulation of public policies aimed at health management on risks associated to drought. The construction of the risk reduction management process, taking into consideration prevention, preparedness, response and recovery, including adaptation strategies, with actions involving community participation would help in increasing resilience by building capacity of the government and of the populations living in this region.

Despite efforts by the government in relation to implementing programs to minimize the impacts associated to lack access of water, food and income, additional mitigation measures are required. Considering the results of this research, it is necessary to implement educational programs in several areas, such as: change in sanitary practices mainly related to the treatment of water for human consumption; food safety; improvement on the use and reuse of water; training of the health sector to ensure appropriate planning for the region focusing on the reality of its drought conditions, in order to provide adequate and equitable health care; and training the population based on their reality of living with semiarid a climate, taking into account their human rights. Other major change processes would be necessary to ensure greater socioeconomic stability in the Semiarid region, such as, basic sanitation, which is extremely necessary to improve the health conditions of the populations; permanent measures of access to water and food; access to education, information and technical knowledge for all; and opportunity to generate employment and income.

Specifically for the health sector, a health professional statement summarizes what we presented in this research: ‘I found interesting this initiative about alerting on drought impacts, because we are already punished and suffer so much from droughts that we have already become accustomed to it, not noticing its impacts’. In view of the results found, some important measures need to be established in order to increase the capacity of management in preparedness and response to droughts. These measures relate to: establishing an intra and intersectoral dialogue to discuss the issue of drought; integrated training with other sectors in risk reduction management associated to droughts; increase knowledge of the risks and vulnerabilities present in each area where the Family Health Strategy operates; improvement in the knowledge of what diseases can be accentuated in drought conditions; development of activities with community participation; improvement in the health information data system, adding variables that could indicate the increase of diseases caused by drought conditions; investigation of the injuries and diseases highlighted by the interviewees, as well as, of the situations of local social and environmental vulnerabilities, in order to subsidize strategies for the prevention of risks and diseases.

The greater perception of the communities living in the Brazilian semiarid refers to the importance of knowing how to live with drought conditions and be resilient, instead of feeling victims or vulnerable to the impacts of drought. In the words of a person from the organized civil society: ‘It is necessary to work with sustainable practices of coexistence with the Semiarid’. This is the main lesson of adaptation, which should be learned by all.

## Competing Interests

The authors have declared that no competing interests exist.

## Data Availability

All relevant data are within the paper. However, to ensure full data availability, the raw data for this paper may be accessed at the Brazilian Observatory of Climate and Health: https://www.climasaude.icict.fiocruz.br/novo/ftp.html. The data can also be accessed through the figshare repository: https://doi.org/10.6084/m9.figshare.7268519.v1 and https://doi.org/10.6084/m9.figshare.7268513.v1.

## Corresponding Author

Aderita Sena: aderitasena@gmail.com
